# Multiple metastases of bones and sigmoid colon after mastectomy for ductal carcinoma in situ of the breast: a case report

**DOI:** 10.1186/s12885-019-6050-1

**Published:** 2019-08-28

**Authors:** Qiuting You, Yichao Fang, Chenchen Li, Yujie Tan, Jianli Zhao, Cui Tan, Ying Wang, Herui Yao, Fengxi Su

**Affiliations:** 1 Guangdong Provincial Key Laboratory of Malignant Tumor Epigenetics and Gene Regulation, Sun Yat-Sen Memorial Hospital, Sun Yat-Sen University, Guangzhou, 510120 China; 2 Breast Tumor Center, Sun Yat-Sen Memorial Hospital, Sun Yat-Sen University, 107 Yanjiang West Road, Guangzhou, 510120 People’s Republic of China; 3 Oncology Department, Sun Yat-Sen Memorial Hospital, Sun Yat-Sen University, 107 Yanjiang West Road, Guangzhou, 510120 People’s Republic of China; 4 Pathology Department, Sun Yat-Sen Memorial Hospital, Sun Yat-Sen University, Guangzhou, 510120 China

**Keywords:** Ductal carcinoma in situ, Breast cancer, Distant metastasis, Metastasis of bone, Metastasis of sigmoid colon

## Abstract

**Background:**

The prognosis of ductal carcinoma in situ (DCIS) is reportedly well. Extremely rare patients with DCIS develop distant breast cancer metastasis without locoregional or contralateral recurrence. This is the first report of multiple bones and sigmoid colon metastases from DCIS after mastectomy.

**Case presentation:**

A 43-year-old woman was diagnosed with DCIS, and she received mastectomy, followed by endocrine therapy and target therapy. During the following-up, convulsions and pain on the legs were complaint. Therefore, Computed Tomography (CT) on bones and positron emission tomography (PET) for whole body were examined in order. Multiple bones and sigmoid colon were under the suspect of metastases, which were then verified by biopsy in the left ilium and colonoscopy respectively.

**Conclusions:**

This case reveals the heterogeneous behavior and the potential poor outcome of DCIS, regular examination and surveillance are necessary even though the distant metastasis rate in DCIS is low.

## Background

Rarely, patients with ductal carcinoma in situ (DCIS) developed distant breast cancer metastasis after mastectomy, the proportion has been reported to be far less than 1% [1, 2]. Even rare are patients with DCIS developing distant metastasis (DM) without preceding invasive locoregional or contralateral recurrence. Therefore, multiple breast cancer metastases of more than one organs after mastectomy for DCIS patients are extremely rare.

We now report our experience with a case of multiple metastases in bones and sigmoid colon after mastectomy for DCIS of the breast.

## Case presentation

In 2016, a unpalpable mass was discovered in a 43-year-old woman on the examination of ultrasound. Excisional biopsy revealed that it was a ductal carcinoma in situ of breast. Modified radical mastectomy for breast cancer was then performed for the patient in GUANGDONG GENERAL HOSPITAL in May 7th, 2016. The postoperative pathological diagnosis was high-grade ductal carcinoma in situ with microinvasion (the largest diameter of invasive region < 0.1 cm), without any lymph nodes involvement. The DICS presented both positive for the estrogen receptor (ER) and progesterone receptor (PR), positive for human epidermal factor receptor 2 (HER2), with a 30% expression of Ki-67. The grade of breast cancer for the patient was characterized as pT1micN0M0.

**Fig. 1:**
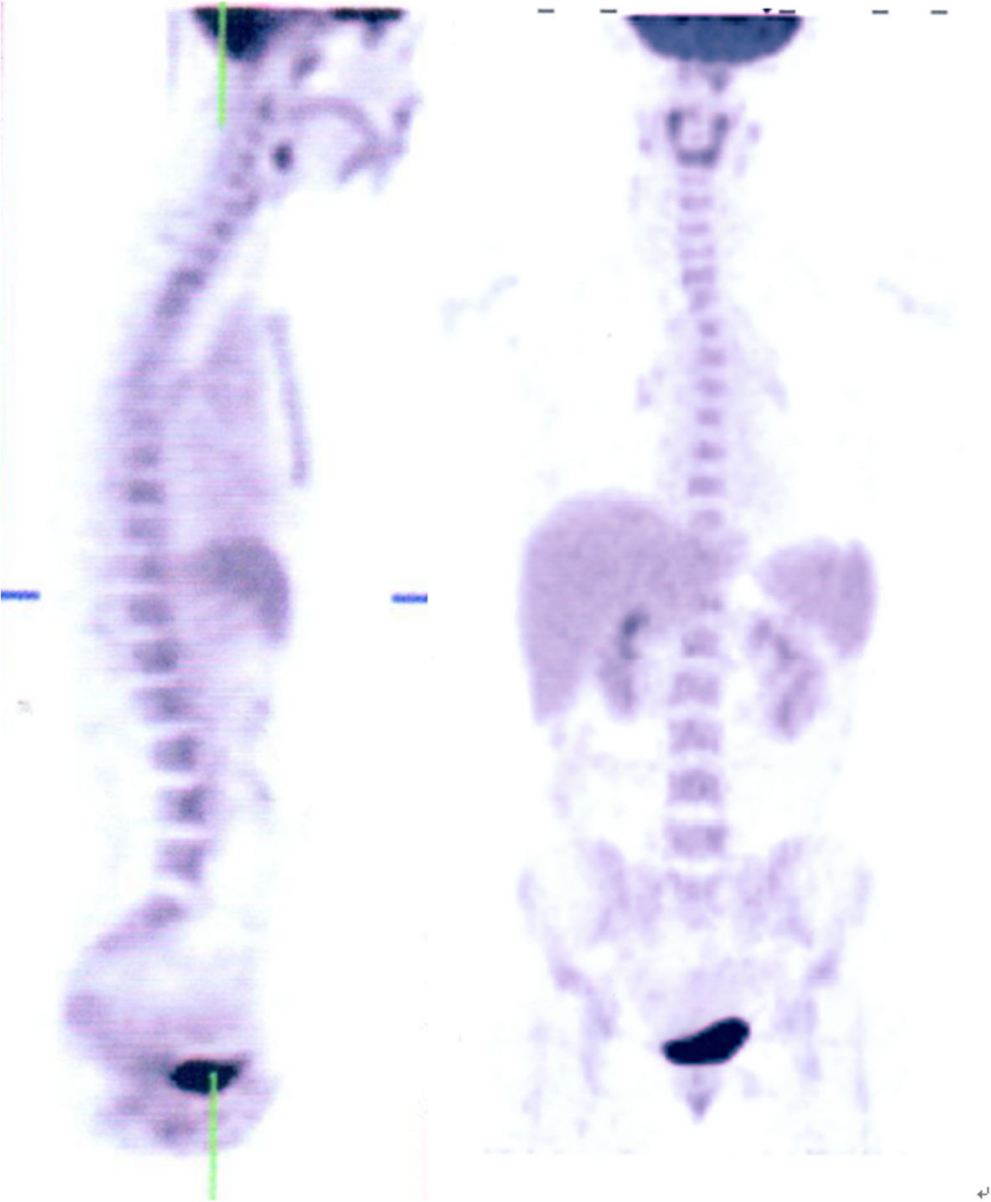
PET/CT revealed abnormal accumulation of FDG in multiple sites in the bone

**Fig. 2:**
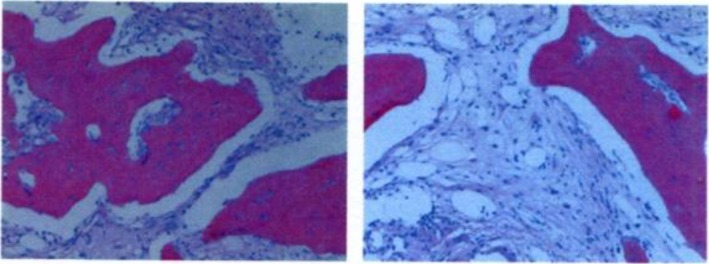
Biopsy of the left ilium at 200× magnification shows metastasis from breast carcinoma

Endocrine therapy and Target therapy were administered for the patient after surgery. Exemestane was consumed 25 mg/day first, but it was replaced by letrozole on January 15th, 2018 due to the shortage of exemestane in Chinese pharmacy. Lapatinib was given 1000 mg/day from June 2016 to June 2017 since a 50% reduction in left ventricular ejection fraction indicting that Herceptin was not suitable on this occasion. Zoledronic acid was used to protect the bones and Leuprorelin was used to suppressed the ovarian function during the same period. On April 12th, 2018, an examination of Computed Tomography (CT) on bones was conducted under the patient’s complaint of convulsions and pain on the legs, the sign of multiple bone metastases were found by the result of CT. Besides, nodes at right abdominal wall and right paracolic sulci region were also under the suspicion of metastases through a further examination by positron emission tomography (PET) (Fig. 1.). Multiple metastases were confirmed by pathological examination after biopsy in the left ilium under CT’s guidance and in the sigmoid colon through colonoscopy (Fig. 2.).

The duration of disease-free survival of the patient was 1 year and 11 months. After the detection of multiple distant metastases, fulvestrant, anastrozole, Leuprorelin and Zoledronic acid were used at the same time to prevent the prognosis of metastatic breast cancer and the progression is stable now.

## Discussion and conclusions

With the confinement of neoplastic lesion to the breast ducts, DCIS is usually precluded by the possibility of DM. Most DCIS patients developing DM had an intervening invasive locoregional recurrence, which was often taken for the prime culprit of the progress. Very rare DCIS patients developed DM and skipped the step of recurrence in former researches. For instance, only 6 of 814 patients developed DM as first event after the diagnosis of DCIS in the National Surgical Adjuvant Breast and Bowel Project B-17 trial [3]. Distant metastases to more than one organs after the initial treatment of DCIS has not been reported previously, this case in our experience is therefore an exception.

The factors associated with the development of DM in DCIS include younger age (<=40 years), positive lymph nodes metastasis, microinvasion, necrosis, negative expression of estrogen receptor, poorly differentiation, preceding or simultaneous invasive locoregional recurrence [2, 4, 5]. Other prognostic pathological markers associated with invasive or noninvasive recurrence and distant metastasis involve positive Her2 expression, high Ki67 staining (> 10%), alone or co-expression [6, 7]. However, due to the small amount of cases with DM after DCIS, these factors didn’t show a meaningful statistical significance in the published series. The presented risks in this case include poorly differentiation, microinvasion, positive Her2 expression, and high Ki67, but the connection between them and DM remains unknown.

DCIS with microinvasion (DCISMi) was defined as DCIS with foci of microinvasion (one or more foci of stromal invasion, none exceeding 0.1 cm in size) by AJCC (American Joint Committee on Cancer) cancer staging manual [8]. However, the interobserver variability of pathologists when assessing the breast specimens might be able to influence the staging and the histopathological features of DCIS [9, 10]. Unnoticeable microinvasion or other histopathological markers might also be omitted or lower assessed in the cancer of this patient in our case.

In terms of pathological characteristics and prognosis, DCISMi resembles closer to stage I breast cancer rather than pure DCIS [11, 12]. Some studies found that patients with microinvasion had a worse prognosis than patients with pure DCIS [11, 13, 14]. The cancer specific death rate for DCISMi patients in Surveillance, Epidemiology and End Results (SEER) database with more than 7 years following-up is 2.4%, which was closer to that of 0.2–1.0 cm invasive breast cancer (1.1% for pure DCIS, 2.4% for 0.2–1.0 cm invasive breast cancer) [11]. Therefore, more rigorous systematic therapy was performed in DCISMi. The rates of mastectomy, post-lumpectomy radiation and chemotherapy were higher for DCISMi than DCIS (40.9, 91.0, 4.1% for DCISMi; 30.6, 80.6, 1.9% for DCIS respectively) [12]. Chemotherapy was not recommended in the guideline of National Comprehensive Cancer Network (NCCN) [15] and the SSO-ASTRO-ASCO DCIS Consensus, both for pure DCIS and DCISMi. However, still 4.1% patients of DCISMi would be treated with chemotherapy, reflecting clinicians’ special attention to the microinvasive portion in DCISMi. This case in our experience is also a warning sign for the potential malignant outcome of DCIS with microinvasion. To some extent, DCISMi should be regarded as invasive breast cancer instead of pure DCIS, the patients with DCISMi should be closely followed-up similar to invasive ductal cancer for the progression of DM.

Most frequent metastatic sites of breast cancer are lung, bone, liver and brain, and different pathological subtypes favor different organs. All the subtypes of breast cancer are inclined to bone metastases, especially in HR+/HER2- and HR+/HER2+ subtypes. The proportions of bone metastases in all DM are 58.52 and 47.28% for HR+/HER2- and HR+/HER2+ breast cancer respectively [16]. While this case in our center metastasizing to multiple bones seemed reasonable since the cancer was HR+/HER2+ subtype, the metastasis to sigmoid colon was quite unusual.

Metastasis to gastrointestinal tract was exceedingly rare for breast cancer and when it happened, sigmoid colon wasnot the most frequent sites for ductal carcinoma [17]. Cases of breast adenocarcinoma with colonic polyp metastasis and ductal carcinoma with scirrhous colonic metastasis had ever been reported in literature [18, 19]. They partly represented the evidence of systemic spread for breast cancer, but no consensus on the metastatic mechanism and proper clinical management could be extracted due to the rarity of gastrointestinal involvement.

Bone metastases in this case were diagnosed by the clinical symptom of bone and joint pain. According to the outcomes in the TEXT (Tamoxifen and Exemestane Trial) and SOFT (Suppression of Ovarian Function Trial) randomized trials, worsening in bone or joint pain was more common in patients with aromatase inhibitor (AI) + ovarian function suppression (OFS) comparing with tamoxifen+ OFS [20]. The pain between bone metastases and side effects of endocrinotherapy is hard to distinguish. Part of the pain in metastatic bones and joints may be mistaken as side effects of AI. Therefore, auxiliary examination by CT and magnetic resonance (MR) is necessary when the clinical symptoms and physical examination could not tell the truth.

This case reveals the heterogeneous behavior and the potential poor outcome of DCIS, which need to be investigated further in the mechanism of metastasis. Tough the distant metastasis rate in DCIS patients is low, regular examination and surveillance in clinical practice are still necessary to detect the unusual event in time.

## Data Availability

Not applicable.

## Abbreviations

AI: Aromatase inhibitor
CT: Computed Tomography
DCIS: Ductal carcinoma in situ
DCISMi: DCIS with microinvasion
DM: Distant metastasis
ER: Estrogen receptor
HER2: Human epidermal factor receptor 2
HR: Hormonal receptor
OFS: Ovarian function suppression
PET: Positron emission tomography
PR: Progesterone receptor
SOFT: Suppression of Ovarian Function Trial
TEXT: Tamoxifen and Exemestane Trial
